# Updating the Mediterranean Diet Pyramid towards Sustainability: Focus on Environmental Concerns

**DOI:** 10.3390/ijerph17238758

**Published:** 2020-11-25

**Authors:** Lluís Serra-Majem, Laura Tomaino, Sandro Dernini, Elliot M. Berry, Denis Lairon, Joy Ngo de la Cruz, Anna Bach-Faig, Lorenzo M. Donini, Francesc-Xavier Medina, Rekia Belahsen, Suzanne Piscopo, Roberto Capone, Javier Aranceta-Bartrina, Carlo La Vecchia, Antonia Trichopoulou

**Affiliations:** 1 Research Institute of Biomedical and Health Sciences, University of Las Palmas de Gran Canaria, and Complejo Hospitalario Universitario Insular—Materno Infantil (CHUIMI), Canarian Health Service, 35016 Las Palmas de Gran Canaria, Spain; laura.tomaino@unimi.it (L.T.); javieraranceta@gmail.com (J.A.-B.); 2 International Foundation of Mediterranean Diet, Nutrition Research Foundation, Barcelona Science Park, 08028 Barcelona, Spain; s.dernini@tiscali.it (S.D.); elliotb@ekmd.huji.ac.il (E.M.B.); alegria.ngo@gmail.com (J.N.d.l.C.); 3 CIBEROBN, Biomedical Research Networking Center for Physiopathology of Obesity and Nutrition, Instituto de Salud Carlos III, 28029 Madrid, Spain; 4 Department of Clinical Medicine and Community Health (DISCCO), Università degli Studi di Milano, 20122 Milan, Italy; carlo.lavecchia@unimi.it; 5 Forum on Mediterranean Food Cultures, 00148 Rome, Italy; 6 Braun School of Public Health, Hebrew University Hadassah Medical School, 91120 Jerusalem, Israel; 7 Human Nutrition, Aix Marseille University, INSERM, INRA, C2VN, 13005 Marseille, France; denis.lairon@orange.fr; 8 FoodLab Research Group (2017SGR 83), Faculty of Health Sciences, Universitat Oberta de Catalunya (Open University of Catalonia, UOC), 08018 Barcelona, Spain; anbachf@gmail.com (A.B.-F.); fxmedina@uoc.edu (F.-X.M.); 9 Food and Nutrition Area, Barcelona Official College of Pharmacists, 08009 Barcelona, Spain; 10 Department of Experimental Medicine, Sapienza University, 00136 Rome, Italy; lorenzomaria.donini@uniroma1.it; 11 Training and Research Unit on Nutrition & Food Sciences, Biotechnology, Biochemistry & Nutrition Laboratory, Chouaib Doukkali University, El Jadida 24000, Morocco; belrekia@hotmail.com; 12 Department of Health, Physical Education and Consumer Studies, Faculty of Education, University of Malta, MSD2080 Msida, Malta; suzanne.piscopo@um.edu.mt; 13 International Center for Advanced Mediterranean Agronomic Studies (CIHEAM), 70010 Valenzano (Bari), Italy; capone@iamb.it; 14 Department of Food Sciences and Physiology, University of Navarra, 31008 Pamplona, Spain; 15 Hellenic Health Foundation, 11527 Athens, Greece; atrichopoulou@hhf-greece.gr

**Keywords:** Mediterranean diet, Mediterranean diet pyramid, sustainable diets, sustainability, environmental concerns, nutrition, food-based dietary guidelines

## Abstract

Background: Nowadays the food production, supply and consumption chain represent a major cause of ecological pressure on the natural environment, and diet links worldwide human health with environmental sustainability. Food policy, dietary guidelines and food security strategies need to evolve from the limited historical approach, mainly focused on nutrients and health, to a new one considering the environmental, socio-economic and cultural impact—and thus the sustainability—of diets. Objective: To present an updated version of the Mediterranean Diet Pyramid (MDP) to reflect multiple environmental concerns. Methods: We performed a revision and restructuring of the MDP to incorporate more recent findings on the sustainability and environmental impact of the Mediterranean Diet pattern, as well as its associations with nutrition and health. For each level of the MDP we provided a third dimension featuring the corresponding environmental aspects related to it. Conclusions: The new environmental dimension of the MDP enhances food intake recommendations addressing both health and environmental issues. Compared to the previous 2011 version, it emphasizes more strongly a lower consumption of red meat and bovine dairy products, and a higher consumption of legumes and locally grown eco-friendly plant foods as much as possible.

## 1. Introduction

The global population is constantly increasing and, according to estimates, from 7.7 billion people worldwide in 2019 it could rise to around 8.5 billion by 2030 and up to 9.7 billion by 2050 [1]. Feeding this global population with healthy food will be a growing concern for governments. Adequate nutrition is not only a survival issue, but is also highly related to the environment and its fragile balance. Food production is the major cause of global environmental change: agriculture occupies about 40% of global land [2], and food production is responsible for up to 30% of global greenhouse gas (GHG) emissions [3] and 70% of freshwater use [4,5]. In other words, diets link worldwide human health with environmental sustainability [6]. Thus, providing an increasing world population with a healthy and sustainable diet represents a major challenge [6]. Food policy, dietary guidelines and food security measures need to move from the traditional approach focused primarily on nutrients and health, to one that takes into consideration sustainability, and its environmental, economic and social dimensions [7]. This also needs to reflect food system factors influencing dietary choices, with their various implications for policy interventions and actions in education, agriculture and the food industry, among others [8,9]. The Mediterranean Diet (MD) has been presented as part of the solution [9,10].

Over the past 50 years, the notion of the MD has undergone a progressive evolution. The limited perception of the MD solely as a healthy dietary pattern [11] has been extended to portray a sustainable dietary pattern embracing the important socio-cultural, economic and environmental benefits of the diet [3,12]. This was done primarily by linking food consumption with production and distribution [13,14], three aspects which are among the main causes of ecological pressure on the natural environment [10]. The MD as a traditional dietary pattern is rich in plant-based foods (cereals, legumes, nuts, fruits, vegetables and herbs) and low in red and processed meat. It includes a moderate intake of fish, seafood, eggs, white meat and dairy products, a moderate intake of alcohol (mainly wine during meals, where culturally acceptable) and olive oil as the main source of added fat [11,14]. Since the 1960s, increasing evidence has shown the protective effect of the MD for cardiovascular diseases, metabolic syndrome, diabetes mellitus, and certain neurodegenerative disorders and cancers [15,16,17,18,19,20,21,22]. However, it has also recently been observed that dietary patterns such as the MD, rich in plant-based foods and low in animal foods are healthier and exert a lower impact on the environment [23,24,25,26].

Indeed, the Mediterranean Diet Pattern (MD-P) has been shown to have a better ecological footprint than current dietary habits in industrialized countries, particularly when compared to the Western dietary pattern [25,27,28,29,30]. This is mainly due to the higher consumption of local and in-season plant-derived foods and lower consumption of animal products. Yet, unfortunately, the current dietary pattern in many Mediterranean countries has shifted from the traditional MD-P. A return to the latter would be beneficial for human health and the natural environment. This can be explained by the fact that the MD is not only a model of cultural food choices, cooking methods, meal patterns and, more broadly, a lifestyle [31,32,33,34]; it is also a sustainable framework that attenuates the environmental pressure of food production and consumption [35,36,37,38,39,40]. A broader adherence to this dietary model would make a significant contribution to greater sustainability of the food system (from producer to consumer), with a myriad of benefits for human and planetary well-being [41].

The transition from the currently consumed diet in most European Mediterranean countries towards a traditional MD-P requires substantial changes in consumers’ values, education and choices. Improvements would also be required in the training of primary care and nutrition professionals [42], as well as changes in agri-food and fishing industry practices, public catering supply, trade policies and areas of research to inform policy and practice. Thus, many stakeholders need to be involved in this change process in order to reach a convergence of the health sector, food production systems and consumer demands. Using a common practical, flexible and science-based graphical guide to illuminate the process would be both efficient and acceptable to all involved.

The aim of the present work is to refine the Mediterranean Diet Pyramid (MDP) with particular attention to the environmental impact of food and based on a consensus between experts in the field. While non-prescriptive from a nutritional (as in specific food) point of view, this update aims to foster the debate about food chain sustainability and to promote the necessary changes for a healthier life, both for humankind and for the planet.

## 2. Materials and Methods

### 2.1. A Historical Overview

The first graphical representation of the (traditional) MDP was developed in 1993 in collaboration with Oldways, Harvard University and the World Health Organization (WHO) [11]. The 1993 MDP was updated in 2009 and 2010 by a group of experts, including one author of the 1993 MDP (AT), as part of this long-standing collaboration. This update was coordinated by the Mediterranean Diet Foundation in collaboration with the Forum on Mediterranean Food Cultures, the Hellenic Health Foundation, the Hebrew University, the International Commission on the Anthropology of Food and Nutrition (ICAF), the Centre International de Hautes Etudes Agronomiques Méditerranéennes (CIHEAM), the Centro Interuniversitario Internazionale di Studi sulle Culture Alimentari Mediterranee (CIISCAM), the Federation of European Nutrition Societies (FENS), the Federation of African Nutrition Societies (FANUS) and the International Union of Nutritional Sciences (IUNS). This review gathered and updated recommendations considering the lifestyle, dietary, socio-cultural, environmental and health challenges that the current Mediterranean populations were facing [14]. The traditional MDP being used at that time was significant as it was intended not only to provide dietary guidance to meet nutritional needs, but also to describe a healthy and sustainable lifestyle using a simple, practical framework which could be adapted to the different cultural and socio-economic contexts of the countries in the Mediterranean region [14]. In 2010, the international symposium “Biodiversity and sustainable diets: united against hunger”, was held in Rome at the Food and Agriculture Organization (FAO), during which a consensus position on a definition of “sustainable diets” was reached, with an entire program session devoted to the MD as an example of a sustainable diet (FAO/Biodiversity, 2012). Subsequently, at the end of 2010, the MD was recognized as an Intangible Cultural Heritage of Humanity by the United Nations Educational, Scientific and Cultural Organization (UNESCO) [31].

**Figure 1:**
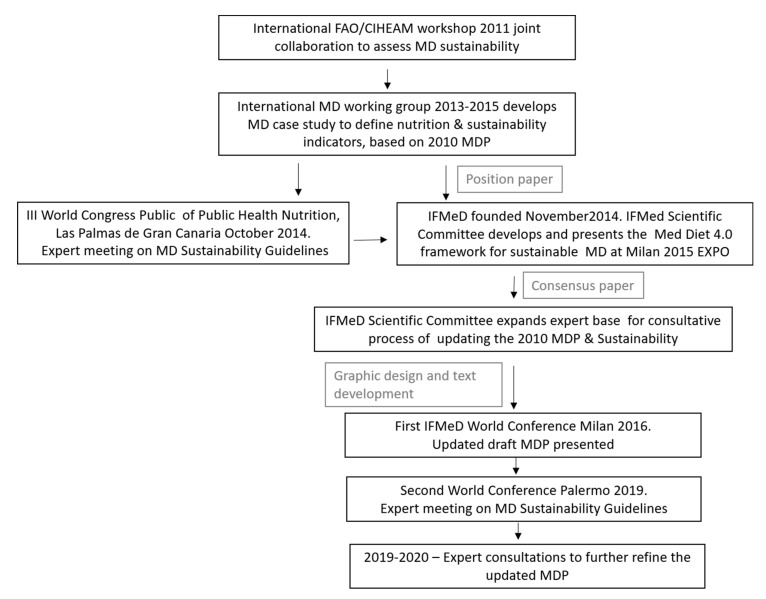
Consensus process for an updated graphical representation of the Mediterranean Diet Pyramid.

After this watershed and historical moment, the MDP continued to be developed as has been outlined in Figure 1. In 2011, an international FAO/CIHEAM workshop was organized to assess the sustainability of the MD model in the Mediterranean region, considering its four dimensions and impacts: health and nutrition, environment including biodiversity, economy, and socio–cultural factors. Initially, a comprehensive list of 74 potential indicators was compiled (FAO/CIHEAM, 2012); this was later reduced to 24 indicators, which was deemed more feasible considering the availability of data sources and the methodological approaches developed. Then from 2013–2015 an international working group from different institutions collaborated to identify 13 nutritional indicators with which to assess the sustainability of a healthy diet, using the MD as a case study and the 2011 MD pyramid model as a reference [43]. In November 2014, the International Mediterranean Diet Foundation (IFMeD) was founded by a group of previous MDP co-authors. The goal was to establish an international center of multi-disciplinary knowledge and expertise, with the objective to revalorize and enhance the MD as a healthy and sustainable lifestyle model, adapting it to current socio-economic and cultural changes, as well as preserving and enhancing it as an intangible cultural heritage of humankind.

As a follow up of these collaborative efforts, the Med Diet 4.0 framework was presented at the Milan 2015 EXPO to valorize the MD as a dietary pattern with multiple sustainability benefits and country-specific variations, embodying the following characteristics: (1) recognized and well-documented major health and nutrition benefits, in the prevention of chronic diseases and in reducing public health costs as well as in the overall improvement of well-being; (2) low environmental impacts and richness in biodiversity, appreciation of biodiversity value, reduction of pressure on natural resources and mitigation of climate change; (3) high positive local economic returns, sustainable territorial development, reduction of rural poverty, and high performance in reduction of food waste and loss; (4) high social and cultural value of food, growth of mutual respect, identity recovery, social inclusion and consumer empowerment. The Med Diet 4.0 framework was released as a joint contribution of the IFMeD scientific committee [13].

IFMeD then continued this consultation process with the goal of establishing an updated consensus position on a newly revised representation of the MD pyramid that incorporated recent scientific evidence highlighting the benefits of the MD as a sustainable and healthy diet. In this exercise IFMeD sought to involve those nutrition, health and socio- culture experts who had been involved in the previous revision of the MDP, as well as new authorities on the subject and a broader expert view. Greater attention was given to environmental issues such as climate change, use of land, use of water, protection of seas and waterways, sourcing and distribution of food, respect for food producers and communication of the value of the MD as a diet in line with scientific environmental recommendations. With this in mind, the first MD world conference “Revitalizing the Mediterranean Diet: from a healthy dietary pattern to a healthy Mediterranean sustainable lifestyle” was organized by IFMeD in Milan on July 2016, and provided a major opportunity to continue this consensus process supporting sustainability and ‘respect’ for the planet. Sustainability and the MD were then addressed more comprehensively in the second MD world conference “Strategies toward more sustainable food systems in the Mediterranean region: the Mediterranean Diet as a lever for bridging consumption and production in a sustainable and healthy way”, organized by CIHEAM-Bari and the Forum on Mediterranean Food Cultures in Palermo, in May 2019.

During the latter half of 2019 and early 2020, further online consultations were held in order to reach consensus regarding an updated MDP. At the same time, the current scientific literature was also examined. The proposed new graphical representation of the MDP responds to the need for a common framework among Mediterranean countries in the form of food-based dietary guidelines [44] which are in line with the 2010 definition of sustainable diets elaborated by FAO [45]. Moreover, it embodies the result of a continuous and rigorous process of consensus building between all relevant stakeholders.

### 2.2. Consensus on an Updated Graphical Representation of the Mediterranean Diet Pyramid

This revision fostered an interdisciplinary dialogue among scientists and experts in public health nutrition, food science, dietetics, social anthropology, sociology, cultural heritage, family and consumer sciences, agriculture, resource management and environmental sciences in order to provide a unified representation of the MD as a sustainable dietary pattern encompassing the entirety of the Mediterranean area. This new revision of the MD Pyramid is created by scientific consensus among experts and is grounded in evidence from research in the fields of nutrition, health and environmental issues. However, it should be emphasized that the MDP described here is not prescriptive. Rather, we suggest that each country uses the basic updated MDP and the recommendations aligned with it as an aid to developing their own guidelines suited to their food systems and culture-rooted cuisines.

## 3. Results

### 3.1. The Updated Mediterranean Diet Pyramid

**Figure 2:**
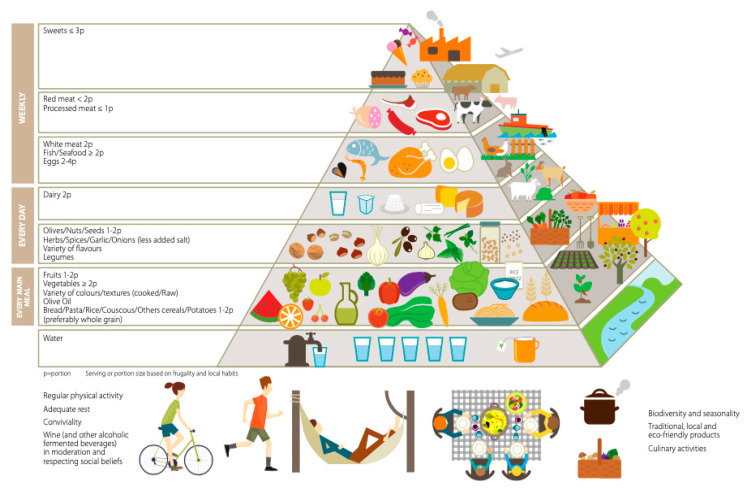
New Pyramid for a Sustainable Mediterranean Diet.

This new graphical representation was conceived as a simplified pyramid framework, to be adapted by different countries in the Mediterranean region to their geographical, socio-economic and cultural contexts, dietary needs and meal patterns. The recommendations target the healthy adult population (18–65 years old) and should be modified to meet the special nutritional needs and diets of children, pregnant women, elderly, and individuals with health problems such as cardiovascular diseases. In this updated pyramid, food items at the base of the pyramid continue to contribute the highest intake levels in terms of grams/day. Animal protein sources are positioned to suggest a lower frequency of consumption and contribution to total intake, having been shifted from daily to weekly consumption. The top of the pyramid presents both animal and sugar-rich foods that should only be consumed occasionally (e.g. red and processed meat, pastries and sweets). Preference for local, seasonal, fresh and minimally processed food is emphasized, supporting biodiversity and eco-friendly and traditional foods. The novelty of the updated MDP lies in its third dimension as shown in Figure 2. This represents the environmental impact of the food items included, as well as aspects of food production sustainability.

### 3.2. Meal Composition

Main meals consumed daily should be a combination of three elements: cereals, vegetables and fruits, and a small quantity of legumes, beans or other (though not in every meal). Cereals in the form of bread, pasta, rice, couscous or bulgur (cracked wheat) should be consumed as one–two servings per meal, preferably using whole or partly refined grains. Vegetable consumption should amount to two or more servings per day, in raw form for at least one of the two main meals (lunch and dinner). Fruit should be considered as the primary form of dessert, with one–two servings per meal. Consuming a variety of colors of both vegetables and fruit is strongly recommended to help ensure intake of a broad range of micronutrients and phytochemicals. The less these foods are cooked, the higher the retention of vitamins and the lower use of fuel, thus minimizing environmental impact.

The highlighted triad of elements for the main meals constitutes the core of the MDP, is based on plant foods, and is responsible for the prevention of numerous chronic diseases and for healthy weight management, as well as for reduced use of natural resources and GHG emissions [22]. Plant foods produced by agro-ecological methods (free from chemical pesticides) can markedly minimize human and nature’s exposure to pesticides [46]. The preference should always be for fresh, seasonal and minimally processed vegetables and fruits. Similarly, choosing local cereal-based products (i.e., bread, couscous, polenta, pasta, rice etc.) when possible and available will support the local economy and reduce the ecologic impact of the production chain.

Agriculture has historically shaped the rich biodiversity heritage of European Union (EU) countries, including those of the South, but over the last decades this synergistic relationship has been undermined [47]. This is why the sustainable management of natural resources is now part of the objectives of the EU’s Common Agricultural Policy agenda, focused on protecting biodiversity and the environment within agriculture [48,49,50]. Food consumption and production have implications for both land use and GHG emissions [51]. In 2017 the total GHG emissions in the EU’s 28 member states (EU-28) were 4483 million tons of CO_2_ equivalents, of which agricultural practices represented around 10% [52]. At the same time, the quantity and type of food consumed directly influences land use. Overall, scientific evidence now supports the general concept that a plant-based diet, compared to the current widely consumed animal food-based diet (especially rich in ruminant foodstuffs), markedly minimizes land, water and resources use for production, along with reducing GHG emissions. Such a diet appears to have significant potential for ensuring food security for all, reducing impacts on climate change and facilitating the realization of the 2030 UN Sustainable Development Goals, as discussed below [53].

However, the nature of this land-food/diet relationship depends on other factors, such as population growth, agricultural productivity, land ownership and investment patterns, as well as land use efficiency [54]. A major limitation to addressing these issues consists of the difficulty in obtaining such estimates for each country.

#### Olive Oil

Olive oil should be the principal source of dietary lipids. Due to its composition and resistance to high temperatures, Extra Virgin Olive Oil (EVOO) is recommended both for cooking and dressing food. Traditionally, in the Mediterranean region, vegetables, other plant foods and staple starchy foods served at principal meals, including pasta, potatoes or rice, are cooked with olive oil, thus amplifying their nutritional value. EVOO has been reported to have a key role in the primary prevention of cardiovascular diseases [18,55] and is inversely associated with certain cancers [56,57,58,59].

Olive production (for use as oil or as olives) represents a significant utilization of land in the southern regions of the EU, particularly Spain (2.4 million ha), Italy (1.4 million ha), Greece (1 million ha) and Portugal (0.5 million ha) [60]. According to the International Olive Council (IOC), the production of table olives in the EU during 2019–2020 was 808.4 (×1000) tons, of which 500 were in Spain, 207 in Greece, 74.1 in Italy and 22.5 in Portugal. Of note, among the other countries of the IOC, Egypt produced 690, Turkey 414, Algeria 300 and Morocco 130 (×1000) tons of table olives [61]. In the olive sector, the negative environmental effects of intensification could be reduced considerably by means of sustainable farming practices. Moreover, with appropriate support, traditional low-input plantations could continue to maintain important natural and social values in marginal areas [60]. On the other hand, several studies [62,63] have shown that olive oil production is associated with adverse effects on the environment during the fruit growth and olive oil production phases. For this reason, it is crucial to identify those phases with greater environmental impacts in order to minimize their effects [62,63]. On a positive note, olive trees are a barrier to desertification and erosion and olive orchards are a CO_2_ sink, removing CO_2_ from the atmosphere and fixing it in the soil. In the production of 1 L of olive oil, olive trees remove 10 kg of CO_2_ from the atmosphere [64].

It is relevant here to discuss palm oil, an industrial alternative to olive oil in the Mediterranean countries. Due to its properties, it is used in many commercial products (e.g., processed foods, cosmetics, biofuels). Globally, palm oil cultivation has increased in the past years, resulting in deforestation (particularly in Indonesia and Malaysia), biodiversity loss, and net GHG emissions. However, Europe remains the leading market for sustainably sourced palm oil, although progress on the number of voluntary initiatives and commitments by industry has been slow [65].

### 3.3. Daily Intake

#### 3.3.1. Olives, Nuts and Seeds

Olives (apart from olive oil), nuts and seeds should be present on a daily basis since they are good sources of unsaturated healthy fats, minerals, vitamins and fiber as well as other compounds with antioxidant potential that contribute to general well-being [66]. Nuts have an important role in the primary prevention of cardiovascular and other diseases [18]. Nuts and olives are high in monounsaturated fatty acids contributing to a desirable monounsaturated/saturated fats ratio. A reasonable consumption (i.e., a handful) of nuts and seeds (minimally salted or unsalted) represent a healthy snack choice, offering plant protein and having good satiety value, amongst others. If possible, locally produced nuts, seeds and olives should be opted for.

#### 3.3.2. Herbs, Spices, Garlic and Onions

Herbs, spices, garlic and onions give dishes flavor, increasing palatability while allowing for a reduction in salt use. They constitute, to different degrees, a source of multiple micronutrients and antioxidant compounds helping to enrich the dishes they are used in. These foodstuffs are staples in many countries in and around the Mediterranean basin and contribute to the regional cultural identities and culinary specialties (e.g., “sofrito” in Italy and Spain and “ladera” in Greece).

#### 3.3.3. Legumes

As indicated earlier, in this updated MDP, preference is given to plant protein sources such as legumes, though animal protein sources low in saturated fats, such as fish, poultry, rabbit and certain lean meats, as well as eggs, are allowed in reasonable amounts. Thus, a daily amount of plant protein sources should be prioritized (at least one small serving per day). Indeed, in this update, legumes have been incorporated into the category of daily consumption. There is no health reason to limit legumes consumption, but there are a number of environmental reasons to increase it. Legumes may substitute animal protein foods in the diet, decreasing the environmental impact of the current MD. Legumes also help to fix atmospheric nitrogen in the soil, improving soil fertility and reducing dependence on energy-intensive or artificial fertilizers. Legumes share some of the health attributes of vegetables with respect to micronutrients and also provide large amounts of protein and soluble fibers. Although of moderate quality, their protein content can be improved if combined with cereals. Legumes are highly satiating with a low glycemic index and glycemic load and the soluble fibers help in controlling blood glucose and cholesterol levels. The versatility of legumes enhances their culinary value. They may be bought dried and then cooked or bought fresh or frozen. Fresh legumes are seasonally available, while frozen and dried versions are available all year round.

#### 3.3.4. Dairy Products

Milk and dairy products should be consumed on a daily basis in a moderate amount (maximum of two servings per day). Traditionally, in the Mediterranean region, the most consumed dairy products were in the form of yogurt and cheese (particularly from sheep’s milk) and these should continue to be consumed in moderation. Dairy products like milk, cheese and yogurt have numerous benefits for bone and muscle health, as they are a source of proteins, calcium and micronutrients. Moreover, due to their probiotic content, they boost digestive tract health and positively affect the microbiome [67,68].

However, dairy products together with meat represent a major concern because of their environmental impact. Dairy farming in the EU is becoming more intensive and more specialized, with imported grains and soybeans being used as feed. There is a move towards fewer and larger farms, except where national authorities actively intervene to help maintain small producers or promote organic production [69]. These trends lead to problems regarding atmospheric, land and water pollution (e.g., from transportation and animal waste) and put pressure on marginal habitats and landscape features, biodiversity and soil integrity. Thus, eating a variety of dairy products and, as much as possible, milk and dairy products from small producers and local farmers should be preferred, as well as consuming organic products. This will help to reduce the environmental impact of these products (i.e., harm to soil quality, or impacts from packaging and transport), sustain the local economy, and yield better quality products, as grazing leads to better lipid profiles in milk.

### 3.4. Weekly Intake

Fish and seafood are integral to the MD and a varied consumption (oily fish, lean fish and shellfish) is recommended based on local availability and culinary traditions. Not only are they important sources of proteins, but Mediterranean Sea fish, such as sardines and others, are rich in omega-3 fatty acids—eicosapentaenoic acid (EPA) and docosahexaenoic (DHA)—reported to reduce the risk of coronary heart disease and to have anti-inflammatory properties [70]. Yet fish and shellfish are largely a wild resource that is at risk of being depleted; therefore, adequate management is needed in order to maintain fish stocks [71]. Apart from seeking sustainably sourced and captured wild fish, aquaculture can be considered as an alternative. Aquaculture fish have equally valid nutritional characteristics, though the lipid profile may be altered due to the feed [72]. Moreover, aquaculture production is now often being redesigned to take on a circular economy approach where waste is reused for other functions, such as feeding plants in aquaponics, or creating energy [73].

Poultry and eggs are also included in the MDP. Poultry provides high quality protein and does not contain the high levels of saturated fat found in red meat. Whole eggs, including those used for cooking or baking, should not exceed four per week. Poultry meat and eggs have moderate impacts on natural resources and environment. Organically produced varieties should be sought, as animal welfare is safeguarded, and this also helps to enrich the soil where these animals roam and deposit waste.

A key recommendation concerns red and processed meats. Red meats should be eaten less frequently (≤2 servings/week), preferably as lean cuts. Similarly, processed meat consumption should also be limited (≤1 serving/week). Both red and processed meats should be seen as a condiment to add palatability to dishes and recipes, and not as the main item in a dish- a common characteristic in the Western dietary pattern. Intake of meat, particularly red and processed meats, has been consistently associated with certain chronic diseases (i.e., increased risk of type 2 diabetes, cardiovascular disease, cancer) and all-cause mortality [74,75]. Thus, a decrease in consumption is beneficial for multiple health reasons. Moreover, this decrease also has high environmental value. In fact, most of the estimated global GHG emissions deriving from agriculture and land use come from livestock production. The process of raising ruminants produces significant amounts of methane, a GHG with detrimental global warming potential. Moreover, livestock production affects land use and GHG emission in different ways: deforestation for grazing land and cropland for soy-feed production, soil carbon loss in pastures, energy required for growing feed-grains and processing and transporting grains and meat, NO releases from the use of nitrogenous fertilizers, and gases from animal manure (especially methane) and enteric fermentation [54,76]. In the future, if acceptability is tackled, alternative protein sources could potentially be in the form of sustainable novel foods such as insects and jellyfish.

Finally, at the top of the MDP, one finds that high fat and/or high sugar sweets, pastries and beverages are represented. Sweets and ultra-processed high sugar, high fat, foods and drinks should be consumed in small amounts and only occasionally. They should be limited to maximum one–two servings per week or reserved for special occasions and celebrations. Sweetness in the diet should preferably be added with fresh and, to a lesser extent, dried fruits, honey or carob syrup. Of note, a lower consumption of highly processed long shelf life sweets, pastries and snacks may contribute to less use by the food industry of palm fats, which are typical ingredients in such foods, and which as described earlier are harmful to the environment unless sustainably produced.

### 3.5. Drinks and Fluid Balance

#### 3.5.1. Water

Water and non-sweetened beverages (1.5–2 L per day, corresponding to an average amount of six–eight servings per day), in addition to water from food are essential to preserve body water balance and maintain an active lifestyle. According to the European Food Safety Authority (EFSA), the reference values for adequate water intake are 2.0 and 2.5 L per day for adult females and males, respectively [77]. These values include drinking water, water from other beverages and water present in food, and apply to individuals engaging in moderate physical activity levels and are at moderate ambient temperature. Water requirements may vary according to age, personal clinical/health status, physical activity intensity, weather and other environmental conditions. Water should be consumed freely, preferably from the tap, according to hygienic safety. Tap water is preferred in order to reduce the environmental footprint of commercial drinking water (mainly packaging and transport). In general, local tap or bottled water should be the order of choice [78] and always prioritizing glass over plastic containers.

#### 3.5.2. Other Beverages

Coffee, tea and herbal infusions (rich in flavonoids) are also included, but consumption should be with a minimal use of sugar or honey, or preferably without any sweetener. Each of these beverages, in varying degrees, offers another source of beneficial flavonoids and can potentially decrease consumption of highly sweetened beverages. Certified fair trade and sustainably produced coffee and tea should be opted for as much as possible, and recipes for local, traditional herbal brews should be recorded for posterity.

### 3.6. Portion Size

Portion sizes (formerly called servings) should be based on frugality and moderation and aligned with the energy needs of urban and modern lifestyles where applicable. In general, the portion sizes of the foods represented at the base of the pyramid should be larger and the foods from this section consumed more frequently (while avoiding food waste). In contrast, those foods at the upper levels should be consumed in much smaller amounts and less frequently.

It is worth noting that in countries adopting the Western diet pattern, most individuals have protein intakes largely exceeding their needs, with red meat being one of the main contributors. Thus, limiting portion sizes of protein-rich foods, particularly red meat, will better fit with a healthy, sustainable diet, and has the added benefit of reducing monetary costs.

### 3.7. The Base of the Pyramid

Situated outside, but at the base of the pyramid, the new concepts of sustainability and affordability are highlighted, adding on to aspects already present in the previous version [14]. Physical activity, adequate rest and socialization during meals are also represented, being practices integral to the definition of the Mediterranean lifestyle.

#### 3.7.1. Physical Activity

The importance of the regular practice of moderate-intensity physical activity (150 min throughout the week, or at least 30 min a day for 5 days per week) and muscle-strengthening activities at least twice a week, are emphasized as a basic complement to the MD for balancing energy intake, maintaining a healthy body weight and for many other health benefits.

#### 3.7.2. Sleep and Rest

A restorative nightly sleep, as well as resting during the day (usually after the mid-day meal) are part of a balanced lifestyle, contributing to health maintenance. A slower lifestyle with reduced stress levels should also be sought. Being active or relaxing in a natural setting (e.g., swimming, walking and hiking, or responsible picnicking) is in keeping with the MD whilst not being detrimental to the natural environment.

#### 3.7.3. Culinary Activities and Conviviality

Mealtimes have a social and cultural value which transcends their nutritional and nourishing functions [79]. Cooking from scratch is typical of the MD, whereas shared culinary activities and shared dining allow for trans-generational transmission of culinary knowledge and recipes in an enjoyable atmosphere. These are key elements for the revitalization of the MD, at least in the Mediterranean region itself.

#### 3.7.4. Wine

As shown by the available scientific literature, there is no safe level for alcohol consumption [80]. Nevertheless, while fully respecting religious principles, cultural beliefs and social norms, an optional moderate consumption of wine (one glass per day for women and two glasses per day for men), preferably during meals, as well as other fermented beverages, might be indicated. In Muslim Mediterranean countries where alcoholic drinks are not consumed, there is a high intake of tea infusions, rich in polyphenols similar to wine, and sometimes accompanied with herbs such as mint. The habit of tea and infusion drinking is commendable from both a health and environmental perspective.

#### 3.7.5. Biodiversity and Seasonal and Local Foods

The value of eating a variety of local and seasonal foods is emphasized. Consuming a range of locally available foods—animal, fish and shellfish and plants—will aid in maintaining biodiversity in the region. As far as possible, fresh, seasonal and minimally processed foods should be preferred. This will maximize their nutritional properties and markedly reduce the environmental footprints of food production and processing, as well as long-distance transport of imported foods. Moreover, local production chains will be sustained, with beneficial effects on the local economy and employment.

#### 3.7.6. Traditional

The selection and preference of traditional and local foods will sustain the local culinary heritage, promote the use of indigenous ingredients, and thus support the (sustainable) production (plants and animal) and capture (e.g., fish, wild rabbit, fowl) of foods which are familiar, and also those less known. Many traditional MD dishes are plant-based and high in vegetables, legumes, nuts and cereals making them more environment-friendly.

#### 3.7.7. Eco-Friendly Products

Consuming eco-friendly products will help the preservation of Mediterranean landscapes and sea. Eco-friendly production methods, such as agro-ecology or organic agriculture, result in biodiversity promotion and reduction or elimination of harmful chemical use [80]. Thus, health for consumers as well as nature (land, rivers, sea, etc.) will be promoted. Indeed, recent large epidemiological studies have shown that consumers whose diet comprises a high share of organic foods adopt a healthier plant-based dietary pattern with lower pesticide exposure, lower body mass, lower impact on natural resources and lower GHG emissions [26,81,82]. Combining the MD with regular organic food consumption appears to be the optimal option [30].

#### 3.7.8. Affordability

Adherence to a MD does not necessarily increase the expense of one’s diet significantly [83,84]. Basing meals on legumes, cereals and local and seasonal vegetables, fruit and fish can help to offset the cost of other potentially more expensive foods such as meat or less healthy processed foods. Following the guidance of the updated MDP can assist those who are financially insecure in consuming a healthier diet.

## 4. Discussion

The seminal EAT LANCET study established a global food system modelling framework to evaluate which combinations of feasible measures (dietary shift, standard and high levels of improved production practices, reduced food waste and loss) were needed to stay within sustainable food production limits (GHG emissions, nitrogen cycling, phosphorus cycling, freshwater use, biodiversity loss and land system change) while still supplying nutritious and healthful diets by 2050. Their findings suggest that a shift towards a dietary pattern comprising more plant-based foods than animal foods (obviating the need to become a strict vegan, and emphasizing fish and poultry, legumes, whole grains, vegetables, fruits and nuts) would provide environmental benefits, nutrient adequacy and improved health [6]. The nutritional adequacy of the traditional MD has been demonstrated, despite being lower in animal products and less caloric, as it contains smaller amounts of proteins and fats and is richer in fiber and micronutrients [11,14,34,43,81].

The proposed new graphical representation of the MDP has considered various aspects outlined by the recent EAT LANCET study and fulfills an expressed need for a common framework among countries in the Mediterranean regions to develop sustainability-promoting Food-Based Dietary Guidelines [38]. It represents a concerted effort at consensus-building among experts from different disciplines, reflective of the complexity of food systems, as well as the need to adhere to the latest scientific evidence.

Assessing the sustainability and especially the environmental impact of the MD has been perceived as a complex task, but one which is urgently required. With this objective, during 2012–2016 an informal international working group from different institutions collaborated to identify nutritional indicators for assessing the sustainability of a healthy diet [43,85]. The group identified thirteen indicators belonging to five areas (biochemical characteristics, food quality, and environmental, lifestyle and clinical aspects). Such indicators were proposed as a useful methodological framework to address health, education and agricultural policies [43]. The significance of the diet for environmental protection or degradation was integral to the choice of indicators, and the role of agriculture and fisheries in facilitating a sustainable diet was considered from different angles.

The role of agriculture has been crucial in historically shaping the biodiversity of EU countries, but over the last decades this synergistic relationship has been undermined. Intensification and specialization of agricultural production has been established increasing production potential, but also causing the marginalization and abandonment of many areas of land and consequently losses of species and habitats associated with farmland [47]. For these reasons, the sustainable management of natural resources is part of the objectives of the EU’s Common Agricultural Policy agenda [48,49,50].

Moreover, changes in food consumption and production could have important implications for land use and GHG emissions [51]. The nature of this land-food relationship depends on the type of food consumed and also on other factors, such as population growth, agricultural productivity, land ownership and investment patterns, and land use efficiency [54].

With the identification of the food production chain as one of the main contributors towards a negative environmental impact in the last decades, the study of such effects has often involved using the Life Cycle Assessment (LCA) method. This method comprises a tool for appraising the environmental impacts and resources used throughout a product’s life cycle. For example, in the case of food production, the LCA investigates the environmental impact of each phase, from agricultural production to final consumption, examining industrial processing, packaging, distribution and retail, cooking and finally waste management [86,87]. Muñoz et al.’s 2010 study analyzed the average Spanish diet (consisting of water 75%, protein 3.6%, fat 5.8%, carbohydrate 13% and fiber 0.78% of ingested food weight) with the LCA method [88]. Results showed that the net Global Warming Potential (GWP) related to feeding a Spanish citizen for one year amounted to 2.1 tons of CO_2_ equivalent. Considering the whole food production chain from production to wastewater treatment, this figure was dominated by the production stage. Moreover, the contribution of meat and dairy production represented around 54% of total GWP for food production. Similarly, eutrophication potential and primary energy use were dominated by the food production stage [88].

The sustainability of the updated MD versus present-day Spanish and Western dietary patterns in the context of the Spanish population was analyzed in 2012–2013 comparing the reference pattern of the MD pyramid [14] with an estimation of the current Spanish and Western dietary patterns derived from FAO data [27]. The MD emerged as having the lowest environmental impact compared to current Spanish and Western dietary patterns, having an agricultural land use of 8365 × 10^3^ Ha/year (Spanish and Western dietary patterns 12,342 × 10^3^ Ha/year and 33,162 × 10^3^ Ha/year, respectively), energy consumption of 239,042 TJ/year (Spanish and Western dietary patterns 285,968 TJ/year and 611,314 TJ/year, respectively), water consumption of 13.2 Km^3^/year (Spanish and Western dietary patterns 13.4 Km^3^/year and 22.0 Km^3^/year respectively) and GHG emissions amounting to 35,510 Gg CO_2_ eq/year (Spanish and Western dietary patterns 72,758 Gg CO_2_ eq/year and 217,128 Gg CO_2_ eq/year, respectively). The food groups mainly responsible for environmental pressure were meat and dairy products [27].

The environmental burden of the MD applied in the Italian context was also analyzed [89]. The authors recognize that the LCA method inevitably suffers from omissions (which are required to make the method applicable) that could lead to underestimation of the total impact when applied to household consumption. For this reason, they chose to assess the environmental footprint of the MD using a hybrid method. This method addresses stages of food production and consumption through the LCA and other methodologies (in this case input-output analysis). It was observed that the national average diet led to 402.91 kg CO_2_ eq/month of GHG emissions, while the MD presented a 6.81% lower CO_2_ eq/month [89]. These results are in agreement with the findings of the study of Muñoz et al. [87], recording slightly higher values of GWP. This is probably due to the authors taking into account the actual consumption of a Spanish citizen, as well as solid waste management, which were not considered in the Italian study.

Two recent studies (2013 and 2018) dealt with sustainability of the MD and dietary patterns in Spain. In the first study, based on the SUN cohort (20,363 adults), it was observed that better adherence to the MD was significantly associated with lower land use, water and energy consumption and GHG emissions, making it an eco-friendly option [28]. In the second study evaluating a cohort of 18,929 adults, the MD was compared to a partly vegetarian diet or Western diet, after ten years of follow-up. Overall, the MD was healthier, whereas the partly vegetarian diet was slightly better than the MD for environmental impacts, whilst the Western diet was better only because of its lower monetary cost. Thus, according to the findings of this study, the MD seemed to be the more sustainable option, closely followed by the partly vegetarian diet [29].

It is important to note (as briefly mentioned earlier) that the environmental impact of food production also involves water use. Water availability is an important issue in some countries of the Mediterranean basin (e.g., in Spain and Malta), linked to the increasing water demand for agriculture plus the desertification process. For these reasons, the concept of a water footprint is gaining importance when analyzing the link between water resources and the food production chain. In the last decades, methodologies such as the Water Footprint Assessment (WFA) and the LCA have been implemented to study such relationships.

The concept of a water footprint (WF) represents an indicator of freshwater use, including both direct water use of a consumer or producer and the indirect water use. In other words, the WF of a product is the volume of freshwater used to produce a food, measured over the whole supply chain. WFA refers to the quantification and location of the WF of a food production chain, and to the assessment of the environmental, social and economic sustainability of this WF. This is carried out with the objective of informing policies and formulating response strategies [90,91].

A study conducted in 2019 [92] investigated the nutritional and water usage implications of the current Spanish diet compared with the MD. The findings showed that the current Spanish dietary habits presented higher WF than the recommended MD: the former consumed 2554 L/capita per day and the latter 1835 L/capita per day. This difference was mainly due to the higher consumption of red and processed meat, sugars, pastries, beverages and dairy products [92]. The authors observed that, in addition to its beneficial effects on health, the adoption of the MD by the Spanish population (approx. 46.6 million) would save 474 million m^3^ of blue water, a valuable resource that could be allocated to other uses. Thus, the MD emerged as a healthy, more sustainable and more water-efficient model than the average Spanish diet. This finding is of major relevance, considering that some of the areas in the Mediterranean region are semi-arid zones.

Widespread international scientific consensus and a large body of evidence support the assertion that plant-based diets are healthier and more protective of natural resources and the general environment, including GHG emissions [11,24,25,93,94]. It is also worth noting that more and more countries are integrating sustainability into their Food-Based Dietary Guidelines (FBDG). For instance, in France the FBDG were extensively updated in 2019 by the Ministry of Health [95]. They now include the concept of environmental preservation and the reduction of pesticide exposure. In brief, they recommend adopting a plant-based diet with increased consumption of all plant foods (vegetables, fruits, whole grains, legumes and nuts) and preferably of organic origin, a reduction in dairy products and limitation of red and processed meats. In an article based on the NutriNet-Santé cohort (28,240 participants), the authors showed that better adherence to the 2019 FBDG was related to higher plant-based food consumption, lower energy intake, lower exposure to chemical pesticides, lower expected population mortality and lower overall environmental impacts (land use, energy demand, GHGs), albeit at a somewhat higher cost [46]. These results suggest that overall, the French 2019 FBDG are generally in line with the multiple dimensions of diet sustainability, including health, although adherence is associated with a slight increase in cost. If adopted by a large part of the population, these dietary guidelines may help to prevent chronic diseases while reducing environmental impacts related to food consumption. The small increase in monetary cost would be balanced by lower externalized costs for the society. This implies that governments should take appropriate measures to help citizens and farmers to adopt sustainable attitudes and practices.

Nevertheless, the evidence supporting the environmental sustainability of the MD has some limitations that should be considered. First of all, there is limited information about each step of the food production chain, and assumptions are sometimes made in environmental impact analyses. Secondly, the food production system is a complex one which requires that many aspects should be controlled and kept in mind. Unfortunately, this remains a challenge as the food production system, transport, distribution and retail are extremely globalized and also diverse. The evaluation of the sustainability of a certain dietary pattern should be context-specific and involve different professionals from the health, medical, sociological and educational fields, as well as from systems engineering, and from agronomic, veterinary and environmental sciences. Moreover, extreme caution must be exerted when discussing the sustainability of food system issues in order to avoid the risk of trivializing problems characterized by extreme complexity, as well as their consequences and/or possible solutions.

Finally, evidence shows that to stay within the guidelines that foster sustainable food systems, a combination of dietary changes and production and management-related measures are required [11,14]. Although putting some of the measures into practice may be sufficient to stay within particular environmental limits, no single intervention is enough to simultaneously meet all the recommended environmental guidelines. This can be seen in the negative effect of climate change on food production, thus emphasizing the need to concurrently address reducing food waste and loss, apart from shifting to more sustainable dietary patterns.

## 5. Conclusions

Past versions of the MDP aimed to describe and summarize the MD patterns of different countries in the Mediterranean area whilst highlighting health benefits or recommendations. The various MD-Ps have all evolved as a result of modern technology, food processing and globalization (e.g., many developments and innovations have changed the range of foods currently available throughout the year). The MD-P is a shared cultural heritage that is widely recognized for its contribution to health and well-being and which should be preserved among the Mediterranean populations. Moreover, according to the United Nations Sustainable Development Goals (SDGs), the MD complies with at least 11 out of 17 goals: SDG2 Zero Hunger: End hunger, achieve food security and improved nutrition and promote sustainable agriculture; SDG3 Good Health and Well-Being: Ensure healthy lives and promote well-being for all at all ages; SDG4 Quality Education: Ensure inclusive and equitable quality education and promote lifelong learning opportunities for all; SDG5 Gender Equality: Achieve gender equality and empower all women and girls; SDG6 Clean Water and Sanitation: Ensure availability and sustainable management of water and sanitation for all; SDG7 Affordable and Clean Energy: Ensure access to affordable, reliable, sustainable and modern energy for all; SDG8 Decent Work and Economic Growth: Promote sustained, inclusive and sustainable economic growth, full and productive employment and decent work for all; SDG11 Sustainable Cities and Communities: Make cities and human settlements inclusive, safe, resilient and sustainable; SDG12 Responsible Consumption and Production: Ensure sustainable consumption and production patterns; SDG13 Climate Action: Take urgent action to combat climate change and its impacts; SDG14 Life Below Water: Conserve and sustainably use the oceans, seas and marine resources for sustainable development; SDG15 Life on Land: Protect, restore and promote sustainable use of terrestrial ecosystems, sustainably manage forests, combat desertification, and halt and reverse land degradation and halt biodiversity loss [53].

The updated edition of the MDP presented here stresses the need to increase the sustainability of the MDP, decreasing the contribution of meat, high fat dairy products and highly processed foods, and increasing the consumption of legumes and as many locally grown vegetables, fruits, nuts and their products, preferably as eco-friendly products, as feasible. The final point to remember is that there should be a comprehensive approach to the MD, promoting it not just as a healthy diet, but also as a culturally coherent way of life to be enjoyed in a sustainable manner. Even if other food models may have a better performance on given points (e.g., certain environmental impacts of plant-based diets) the overall value of the MD on four dimensions, that is environmental, social, cultural, economic and nutritional/health levels, makes it a sustainable diet model that has been also recognized as an intangible heritage of humanity [3], but with added environmental value. The virtues of the MD and particularly its significance for environmental preservation and protection as presented in this new version of the MD pyramid need to be efficiently communicated and applied in an integrated manner at multiple levels. This implies starting from the level of policymakers, and expanding to mass media and social media influencers, to community development NGOs, to educators, to food innovators in production and processing, to restaurateurs and finally to the individuals and families in households.

This revision was fostered by an interdisciplinary dialogue amongst a multidisciplinary team of scientists and experts in order to provide a unified representation of the MD as a sustainable dietary pattern relevant to the entire Mediterranean region. This new revision of the MD Pyramid is grounded in scientific consensus among experts, as well as in evidence from research in the areas of nutrition, health and the natural and socio-economic environments; but it does not intend to be prescriptive. The idea is that each country uses the basic updated MDP and related recommendations as a guide, adapting the contents to their own country-specific contexts and cuisines.

This updated representation of the MD pyramid is released in order to marry the worldwide interest in the MD with increasing attention to sustainability, and especially to environmental concerns. Such representation aims to contribute towards a significantly greater adherence to this dietary pattern and lifestyle in the Mediterranean basin, as well as in other similar countries. The main goal is to shift the perception of the MD benefits from a person-centered, individual focus, to a broader focus embracing the benefits of the MD pattern for the planet and its populations.
